# Antibiotic use and impact on illness course in children with influenza-like-illness in the emergency department

**DOI:** 10.1017/ash.2022.103

**Published:** 2022-05-16

**Authors:** Nicole Poole, Sean O’Leary, Suchitra Rao, Krithika Suresh, Angela Moss

## Abstract

**Background:** Child absenteeism from childcare or school leads to economic loss from parental work absenteeism, overutilization of acute-care resources, and excess medicalization of children with minor illnesses. We sought to determine the difference in days missed from childcare or school and days of illness for children with influenza-like illness (ILI) in the emergency department (ED) who are or are not prescribed an antibiotic. **Methods:** We conducted a secondary data analysis of a prospective randomized control trial evaluating the impact of rapid molecular testing on provider decision making. The study included children aged 2 months–12 years attending childcare or school seen in the ED from December 2018 through December 2019 with ILI (CDC definition) with parental survey completion 10 days after their ED visit. The primary exposure was receipt of antibiotics over the course of illness, which was assessed by chart review and parent survey. The primary outcome was number of days missed from class. The secondary outcome was number of days of illness after initial ED visit. Wilcoxon tests were used to compare missed class days or illness days by antibiotic receipt. Multivariable negative binomial regression was used to analyze outcomes, controlling for clinically important patient characteristics. **Results:** Of 251 children included in this study, the median age was 4.2 years (IQR, 1.6–7.0); 52% were male, 40% were White, 54% were Hispanic, and 75% had government insurance. Antibiotics were prescribed in 26% of ILI encounters. There was no statistically significant association between antibiotic receipt and number missed class days (2.0 days [IQR, 1.0–4.0] vs 3.0 days [IQR, 1.0–5.0]; *P* = .08) or illness days (4.0 days [IQR, 3.0–7.0] vs 5.0 days [IQR, 3.0–7.0]; *P* = .13) after the initial ED visit. After adjusting for covariates, there was no significant difference in missed class days or illness days for patients prescribed antibiotics in relation to days sick before ED visit. The rates of missed class days and illness days were 87% and 30% greater, respectively, in patients with additional medical visits during the course of illness. **Conclusions:** Days sick prior to ED presentation and receipt of an antibiotic for ILI had no influence on child absenteeism or illness duration. However, children missed more class and received more antibiotics if they had multiple medical visits during an illness. Further study is needed on sociobehavioral factors leading to medicalization of children with minor illnesses and its impact on the unnecessary use of antibiotics.

**Funding:** None

**Disclosures:** None

